# A Financial Benefit-Cost Analysis of Advancing Jordan's Medical Tourism: The Case of Proton Therapy

**DOI:** 10.7759/cureus.77119

**Published:** 2025-01-08

**Authors:** Ahmad N Fasseeh, Emad Almomani, Baher Elezbawy, Youssef H Ahmed, Kareem A El-Fass, Emad Alsharu, Zoltán Kaló

**Affiliations:** 1 Health Economics, Syreon Middle East, Alexandria, EGY; 2 Health Economics, Jordanian Royal Medical Services, Amman, JOR; 3 Evidence Synthesis, Syreon Middle East, Alexandria, EGY; 4 Reproductive Endocrinology, Jordanian Royal Medical Services, Amman, JOR; 5 Health Economics, Syreon Research Institute, Budapest, HUN

**Keywords:** benefit-cost ratio, cost-benefit analysis, internal rate of return, investment, jordan, medical tourism, net present value, proton therapy

## Abstract

Objectives

Jordan is moving forward toward enhancing medical tourism. Proton therapy (PT), an effective intervention for cancer treatment, is unavailable in some countries, often necessitating international travel to receive treatment. This study aims to evaluate the cost-benefit of establishing a PT facility in Jordan to address this gap. Our study is one of the first studies to evaluate the economic impact of medical tourism through establishing a PT facility, showing its potential to position Jordan as a regional hub for advanced medical care.

Methods

We conducted a financial benefit-cost analysis using an ex-ante analysis model. The analysis was conducted from two distinct perspectives: the healthcare payer perspective and the societal perspective. Model inputs were derived through a literature search and questionnaires completed by local experts. Deterministic sensitivity analysis (DSA) and probabilistic sensitivity analysis (PSA) were used to assess model robustness and explore key uncertainties. To overcome uncertainty in projected user numbers, two scenarios were created: a conservative scenario with average capacity, and an optimistic scenario based on maximum device utilization.

Results

The benefit-cost ratio (BCR) of establishing the facility was 1.24 from the societal perspective. This perspective showed a net present value (NPV) of 15 million Jordanian dinars (JOD), an internal rate of return (IRR) of 12%, and a payback period of seven years. From the public payer perspective, values were less favorable, but still positive, with a BCR of 1.08, an NPV of 5 million JOD, an IRR of 6%, and a payback period of nine years. Scenario analyses showed that, if the device is utilized to its maximum capacity, the benefit would increase reaching BCRs of 1.80 and 1.58 from the societal and healthcare payer perspectives, respectively. DSA and PSA proved the robustness of the model results.

Conclusions

The model shows that establishing a PT facility in Jordan is cost-effective, and is expected to generate substantial economic returns, making it a justifiable investment decision.

## Introduction

Proton therapy (PT), an innovative technique in the field of radiation treatment, has a crucial role in combating a variety of cancers [1]. Unlike traditional radiation therapy using X-rays, PT administers a focused beam of protons to target cancerous tissue. This level of precision is particularly advantageous in treating tumors located in delicate or inaccessible areas, such as the brain or spine [1].

PT also significantly lowers the radiation dose received by non-targeted tissues compared to conventional photon therapy, thereby reducing the risk of radiation-induced secondary malignancies [2].

Specifically for pediatric patients, PT has become vital due to its potential to minimize long-term complications due to radiation damage in growing tissue. This is of paramount importance considering the large number of children diagnosed with cancer each year, with CNS malignancies accounting for approximately 21% of these cases [3].

However, PT’s advantages come with financial implications. The huge investment required to purchase a PT device increases the treatment costs per patient to three times higher than the standard of care. Besides the investment costs, there are also high operating costs and maintenance fees [4].

PT device is an expensive intervention that is available only in a limited number of countries worldwide. Currently, no PT facilities are available in Jordan or its neighboring countries [4,5]. In early 2023, only 89 PT facilities were available worldwide [6]. Therefore, patients requiring PT may need to travel to receive their treatment sessions. This is referred to as medical tourism [7]. Patients travel to other countries for treatment for one of two main reasons, either to receive a cheaper treatment or to receive a treatment that is not available or approved in their countries [7].

Over the years, Jordan has seen a rise in medical tourism, primarily because of the high caliber of care and experience Jordanian hospitals and medical personnel offer. The International Medical Travel Journal (IMTJ) demonstrates the growing interest in Jordanian healthcare and medical tourism [8].

Jordan is treating patients from the Gulf region, Saudi Arabia, Yemen, Iraq, Syria, Libya, and many other countries [9]. The country has made significant investments in hospital infrastructure and staff training, demonstrating a strong commitment to enhancing its healthcare services as a part of a broader national objective to generate significant revenue for the Ministry of Health, targeting a one billion dollar revenue [10]. These efforts have positioned Jordan as a medical tourism destination, currently ranking first in the Middle East and fifth worldwide [10], making it a potential candidate for establishing a PT facility in the region.

The financial feasibility of investing in a PT facility in Jordan faces significant challenges due to the country’s small population and the specific eligibility criteria for PT treatment. PT is primarily indicated for specialized cases, such as pediatric cancers and certain brain tumors, which significantly narrows the potential patient base [11,12]. As a result, relying solely on domestic cases may not generate a sufficient patient volume to justify the feasibility of investment. Consequently, the high cost and restricted utilization may prevent the technology from being cost-beneficial for the local demand [4,11,12].

Medical tourism can increase the number of patients using the PT device, and it could be therefore considered cost-beneficial. However, the benefit-cost ratio (BCR) of PT should be calculated before making a decision.

Economic analysis studies have shown that PT can be cost-effective compared to other treatments [13]. However, the available literature is usually concerned about the cost-effectiveness of the treatment per patient, not the cost-benefit of the investment itself. Additionally, findings of published studies are typically specific to certain medical indications, and efficacy of the device against other comparators.

There is a notable gap in the cost-benefit of such a facility, both in Jordan and globally. Additionally, studies considering the impact of medical tourism on PT are scarce.

Recognizing this gap, we aim to provide an estimate of the financial benefit-cost of establishing a PT facility in Jordan from a societal (holistic economy of Jordan) and from a public payer (Jordanian Royal Medical Services (RMS)) perspective. We focus not only on the financial outcomes but also on the broader implications for healthcare and medical tourism. The study findings offer a thorough understanding of the economic viability and potential benefits of PT in Jordan from different perspectives to contribute to informed policy decisions in Jordan.

## Materials and methods

We created a comprehensive ex-ante analysis model tailored to support informed decision-making regarding investment in PT technology with a special focus on enhancing medical tourism in Jordan. The model estimates the total costs and revenues associated with the PT device over its lifespan, to determine if the investment provides good value for money. Here, we outline the model, detailing its structure, drug costs, revenues, assumptions, and uncertainty.

Model design

The model of choice was a financial benefit-cost analysis. The intervention is the acquisition and operation of a PT facility. This is compared to the alternative situation of not acquiring or operating such a facility.

It is important to note that this analysis does not aim to evaluate the cost-effectiveness of PT compared to conventional therapies, but rather to explore its potential return on investment through enhanced medical tourism and broader economic contributions.

Target population

The target patient population includes three distinct groups: eligible local Jordanian patients, eligible international patients, and Jordanian patients who would typically receive PT treatment abroad. The target patient number estimation was derived from an abstract presented at the 2023 European Society of Radiotherapy and Oncology (ESTRO) conference by Akash M. and Salem A., which reported the number of patients eligible for PT in the Middle East, including Jordan [14]. We validated these data through local datasets and expert questionnaires.

The abstract reported the proportion of pediatric cancers in Jordan (2.9%) and estimated the annual eligible population for PT at 534 patients, including 139 pediatric and 395 adult cases [14].

To validate these data, we searched local publications. The 139 pediatrics and 395 adult patient values that we used aligned with the data from King Hussein Cancer Center (KHCC) and the Jordan Cancer Registry (JCR), respectively [15,16].

Between 2011 and mid-2022, KHCC registered 2,779 Jordanian children with cancer. Non-leukemia pediatric cancers accounted for 4.3% of total cancer cases in Jordan, with 217 pediatric cases reported in 2012, including brain tumors and lymphomas [15,16].

Similarly, adult cancer data from JCR highlights prevalent types such as breast and colorectal cancers, supporting the estimate of 395 adult cases. These figures we used, while consistent with the ESTRO data, still required validation from local experts [15,16].

To further validate these, we created a structured questionnaire (Appendix Table 6) and distributed it to Jordanian healthcare experts who confirmed the validity of those values.

For the international patient cohort, conservative estimates were derived from published data on Egypt, Saudi Arabia, and Kuwait. This approach likely underestimates the actual patient influx, as it does not account for potential patients from other countries seeking treatment in Jordan. These countries were considered due to their geographical proximity to Jordan and the absence of proton therapy devices in their healthcare infrastructure [17,18]. Table 1 below presents the estimated patient population based on the ESTRO conference abstract [14], illustrating the potential reach and impact of PT within and beyond Jordan.

**Table 1:** Estimated eligible patient population. Source: Akash and Salem [14].

Patient population	Jordan	Kuwait	Egypt	Saudi Arabia
Pediatric cancer excluding leukemia	139	34	1912	384
Additional adult cancer patients	395	98	2756	643
Total	534	132	4668	1027

These values represent all potential patients from these countries. However, our model assumes that only a small proportion of these patients will be treated in the facility in Jordan to be conservative. The market uptake was calculated by integrating the market share of PT and device accessibility to ensure a realistic representation of the potential patient base.

Perspectives

We conducted the model from two distinct perspectives to thoroughly assess the economic feasibility of PT in Jordan.

For the societal perspective, we adopted a holistic economic perspective of Jordan to assess the potential impact of PT as a driver for medical tourism, focusing on its return on investment for Jordan’s economy. This perspective incorporated both medical and non-medical revenues offering an expansive view of PT’s economic impact.

From the public payer perspective, we adopted the perspective of the Jordanian Royal Medical Services (RMS), focusing exclusively on the direct medical revenues and costs associated with PT. This approach provided insights into the direct financial implications for the healthcare system.

Scenarios

For each perspective, we adopted two alternative scenarios: a conservative scenario for the base case, and a maximum capacity (optimistic) scenario. The results of these scenarios were calculated to reduce uncertainty regarding the estimated number of device users. The two-scenario approach aims to provide decision-makers with a range of outcomes to plan with a defined range of possibilities.

For the conservative scenario, we calculated the results based on only 5% of the eligible population being treated with the device. However, the real utilization could be more. Therefore, we created a maximum capacity scenario that includes the maximum number of patients who could benefit from the device while keeping the same parameters, such as time horizon and number of rooms.

Time horizon

The model’s time horizon was assumed to be the same as the estimated device life span, which was set conservatively for 10 years, chosen for relevance to the budgeting process of stakeholders according to previous studies [19].

Input data

Device base case parameters, healthcare costs, and resource utilization were estimated from local sources. We disseminated a questionnaire to local healthcare experts in Jordan (Appendix Table 6), including a panel of carefully selected local experts with extensive professional experience in oncology and health economics. The panel included local healthcare experts in addition to a health economist from the Jordanian Ministry of Health and Jordanian RMS with significant expertise in economic evaluations and financial models for healthcare interventions. This expertise was instrumental in formulating key cost and resource utilization parameters. Additionally, the panel included a medical specialist who is the head of the health technology assessment unit in RMS, who provided critical clinical insights into patient care pathways and treatment eligibility for PT. The expert contributions ensured that the analysis aligned with local clinical practices and cancer management protocols, enhancing the study’s relevance and accuracy.

Costs associated with the PT facility were stratified into three primary categories: fixed costs, variable costs, and marketing costs. Fixed costs comprised one-time expenditures critical for establishing and maintaining the facility, including construction, equipment, imaging devices, training, insurance, and ongoing maintenance.

Variable costs represented operational expenses that vary with patient volume, covering medical staff salaries, administrative and support team wages, electricity, consumables, and other operational necessities. Marketing costs were allocated to promote the facility, aiming to attract medical tourists and enhance the center’s visibility within the healthcare sector.

Data on non-medical expenditure, such as food, accommodation, and other living costs were collected from the literature and validated through the experts' questionnaire. These costs were collected specifically for patients and their companions on a monthly basis. These data were then adjusted to a daily value to accurately reflect per diem revenues associated with medical tourism. These non-medical costs are essential in understanding the broader economic implications of PT for Jordan, as they contribute significantly to the overall spending associated with international patient stays.

Other required inputs were assumed or estimated from the published literature. Tables 2, 3 below present the values and sources of model parameters and related costs, with the full expert questionnaire responses available in Appendix Table 7.

**Table 2:** Device parameters. LoS: length of stay; kWh: kilowatt per hour; IT: information and technology.

Parameters	Value	Source
Number of rooms (gantries)	2	Jordan experts questionnaire
Maintenance costs start at year (before the selected year, maintenance costs are covered with the device’s cost based on the contract)	8	Jordan experts questionnaire
Device capacity (patients per day per room)	25	Jordan experts questionnaire
Device working days per year	275	Jordan experts questionnaire
Sessions required per patient	20-35	Jordan experts questionnaire
Average length of treatment in days per patient	32	Texas Center for Proton Therapy [20]
Average length of stay (LoS) of international patients in days in Jordan	34	Assumption
Physicians (radiation oncologist) required per room	4	Jordan experts questionnaire
Nurses required per room	4	Jordan experts questionnaire
Nurse aids required per room	4	Jordan experts questionnaire
Radiation therapist required per room	12	Jordan experts questionnaire
Dosimetrists required per room	2	Jordan experts questionnaire
Medical physicists required per room	4	Jordan experts questionnaire
Anesthesia specialists required per room	2	Jordan experts questionnaire
Secretary required	2	Jordan experts questionnaire
Information and technology (IT) specialist support required	1	Jordan experts questionnaire
Financial department employees required	2	Jordan experts questionnaire
Human resources (workers) required	10	Jordan experts questionnaire
Maximum number of patients per year	491	Calculated

**Table 3:** Device-related costs. JOD: Jordanian dinar.

Device-related costs	Value	Source
Equipment and installation per room	JOD 22,500,000	Jordan experts questionnaire
Other equipment	JOD 15,000	Assumption
Consumables per patient	JOD 85	Jordan experts questionnaire
Maintenance cost per device (including 2 gantries (annual))	JOD 800,000	Jordan experts questionnaire
Training (annual)	JOD 0	Jordan experts questionnaire (Covered in the contract)
Marketing (annual)	JOD 2,000	Assumption
Device electricity usage per treatment course per patient (kWh)	52	Dvorak et al. [21]
Cost of electricity per kWh	JOD 0.087	Global Petrol Prices website [22]
Medical staff costs (salary per month)		
Physician (radiation oncologist)	JOD 4,000	Jordan experts questionnaire
Nurse	JOD 1,500	Jordan experts questionnaire
Nurse aid	JOD 600	Jordan experts questionnaire
Radiation therapist	JOD 800	Jordan experts questionnaire
Dosimetrist	JOD 1,000	Jordan experts questionnaire
Medical physicist	JOD 800	Jordan experts questionnaire
Anesthesia specialist	JOD 800	Jordan experts questionnaire
Non-medical staff costs (salary per month)		
Secretary	JOD 450	Jordan experts questionnaire
Information and technology (IT) support specialist	JOD 600	Jordan experts questionnaire
Financial department employee	JOD 800	Jordan experts questionnaire
Human resources (workers)	JOD 300	Jordan experts questionnaire
Treatment abroad		
Cost of treatment outside Jordan	JOD 50,000	Assumption

The analysis was conducted based on a PT device with two gantries, following the recommendation from the experts. Regarding device maintenance costs, it was set to start in year eight in the base case (i.e., based on the contract, the purchaser needs to pay for maintenance starting from year eight, while in previous years, maintenance costs are covered by the manufacturer). The average length of stay in Jordan for international patients was assessed and assumed by experts according to treatment duration.

All costs were obtained from local Jordanian experts, except for electricity and the cost of treatment abroad for Jordanians. Electricity costs were calculated from a study reporting usage of PT per treatment course per patient in kWh [21] and then multiplied by the Jordanian electricity price in the year 2021 for industrial consumption [22]. Other costs, equipment, marketing, and treatment abroad, were assumed. For validation, unit costs of medical and ancillary staff in Jordan were cross-referenced with findings reported by Hammad et al. in a study published in 2022 [23]. All costs were collected for the year 2023.

Number of patients for maximum capacity scenario

To determine the maximum number of patients per year, the number of rooms was multiplied by the device’s daily patient capacity and device working days and then the total was divided by the average number of sessions required, as shown in the equation below.

(Number of rooms * Capacity of patients per day * Device working days per year)/Average number of sessions per patient

Revenues

The model identified three main revenue streams: tourists' medical expenditure, tourists' non-medical expenditure, and prevention of cash outflow. Tourists' medical expenditure reflects revenue generated from treatment fees paid by international patients. Tourists' non-medical expenditure accounts for additional spending by patients and companions on accommodation, food, and other services, contributing to the local economy. Preventing cash outflow refers to the economic benefit of reducing the need for Jordanian patients to seek PT abroad, thereby retaining healthcare spending within the country. Local patients’ medical expenditures were not included as revenues, as they are already paid from the government’s budget.

The intended price per patient for proton therapy was set at approximately 35,000 USD by the experts. This pricing is set to be consistent for the entire course of treatment, irrespective of the number of sessions, as it reflects the comprehensive service provided.

The total benefit was calculated based on three main components: treatment revenue from international patients, non-medical spending, and prevented cash outflow. The calculation is shown below.

Number of patients * Number of sessions * Cost per session + Number of days * (Number of tourists + Number of companions) * Nonmedical expenditure per day + Number of Jordanians who would have traveled for proton therapy * Cost of traveling for proton therapy

Total costs encompassed establishment costs, ongoing maintenance, staffing, power, and variable costs per session, as shown below in the equation.

Establishment cost (construction + equipment) + Maintenance + Staffing + Power + Number of patients * Average number of sessions per patient * Variable cost per session + Marketing expenditure

The net present value (NPV) was calculated as the difference between total benefits and costs, shown in the equation below presenting the incremental economic value associated with establishing a PT facility in Jordan.

NPV = Total Benefit - Total Cost

Uncertainty

The model enables decision-makers to interpret the uncertainty in outcomes by applying deterministic sensitivity analysis (DSA) and probabilistic sensitivity analysis (PSA) via built-in tools for all model inputs, and the effect on NPV was assessed. To assess the robustness of the model results, DSA was conducted by varying one model input or assumption one at a time. Each model parameter was varied by its 95% CI if such information was reported in the original source. The modeled parameters were varied by ±10% of the base-case value if the 95% CI was unavailable.

PSA was conducted to estimate the random variability in model inputs. A Monte Carlo simulation with 1,000 iterations was conducted. In each iteration, the model inputs were randomly drawn from the specified distributions. Whenever available, the standard error (SE) of the selected distribution was obtained directly from the same data source that informed the mean value. In the absence of data on the variability around health state cost values, variability was assumed as 25% of the mean value. The list of varied model inputs and their distributions are provided in Appendix Table 8.

## Results

The model results are formulated into four main outcomes: BCR, NPV, internal rate of return (IRR), and payback period. Each outcome is reported in the base case (conservative) scenario and in the maximum capacity (optimistic) scenario for both perspectives.

Base-case results

While both the societal and public payer perspectives demonstrate promising financial results, there are some disparities. From the wider Jordanian economy perspective, the BCR is 1.24, the NPV is 15,407,893 Jordanian dinars (JOD), and the IRR is 12%. If only medical services revenues are considered (public payer perspective), the BCR is lower at 1.08, NPV is 5,178,724 JOD, and the IRR is also lower at 6%. For the payback period, the Jordanian economy perspective results showed a period of seven years, while the RMS perspective showed nine years. Table 4 shows the results of the base case for both perspectives.

**Table 4:** Base case results. JOD: Jordanian dinar.

Outcomes	Jordanian economy perspective (medical and non-medical revenues)		Royal Medical Services perspective (only medical revenues)	
	Value	Interpretation	Value	Interpretation
Benefit-cost ratio (BCR)	1.24	For each JOD spent, the project returns 1.2 JOD	1.08	For each JOD spent, the project returns 1.1 JOD
Net present value (NPV)	15,407,893	The project returns 15,407,893 JOD	5,178,724	The project returns 5,178,724 JOD
Internal rate of return (IRR)	12%	Annual rate of growth	6%	Annual rate of growth
Payback period	7	The payback period is in year 7	9	The payback period is in year 9

Uncertainty analysis results

**Figure 1:**
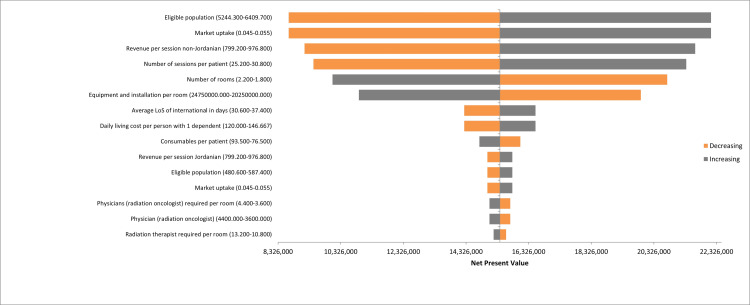
Deterministic sensitivity analysis (DSA) Tornado diagram showing the highest impact inputs on the NPV. NPV: net present value; LoS: length of stay.

DSA illustrates the sensitivity of the NPV to numerous factors. It shows the 15 most influential variables when base-case estimates of the input parameters were varied by ±10% (Figure 1). The figure shows that the model is most sensitive to changes in the number of eligible populations, market uptake, revenue per session, and number of sessions per patient. However, varying these by 10% does not cause the NPV to reach negative values.

**Figure 2:**
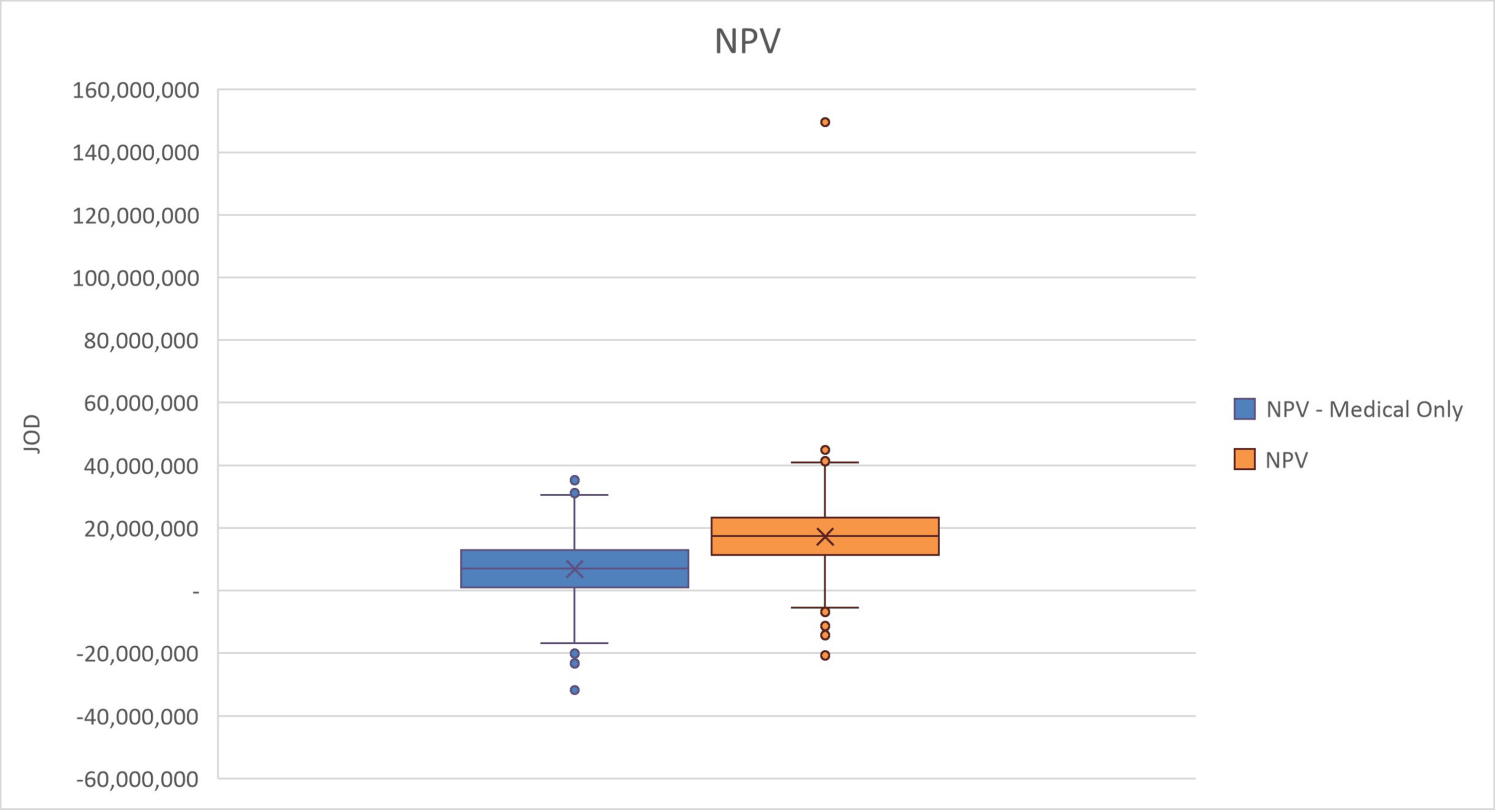
Probabilistic sensitivity analysis (PSA) box and whisker plot showing the net present value (NPV) of investment in the device. JOD: Jordanian dinar.

**Figure 3:**
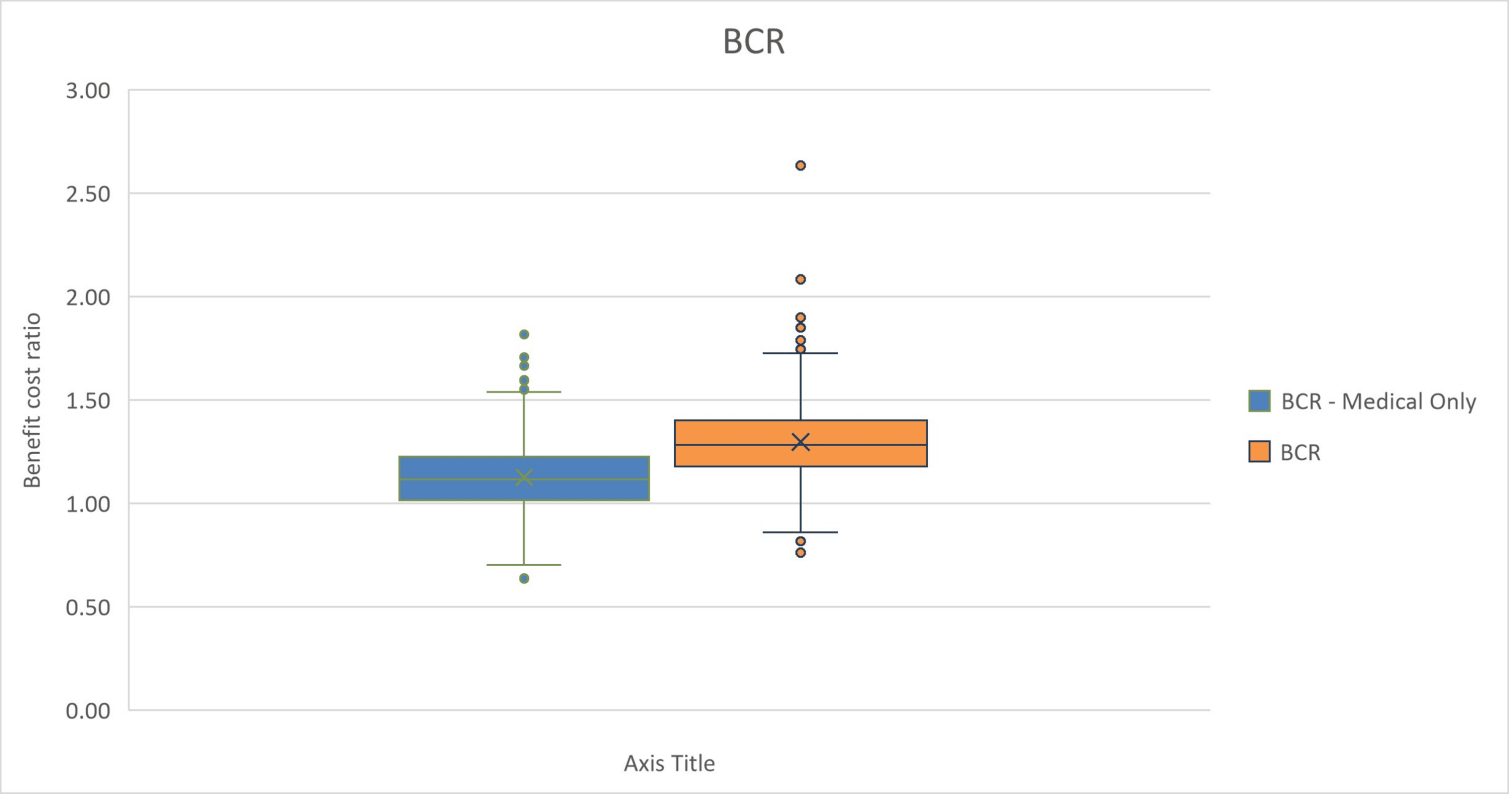
Probabilistic sensitivity analysis (PSA) box and whisker plot showing the benefit-cost ratio (BCR) of investment in the device.

**Figure 4:**
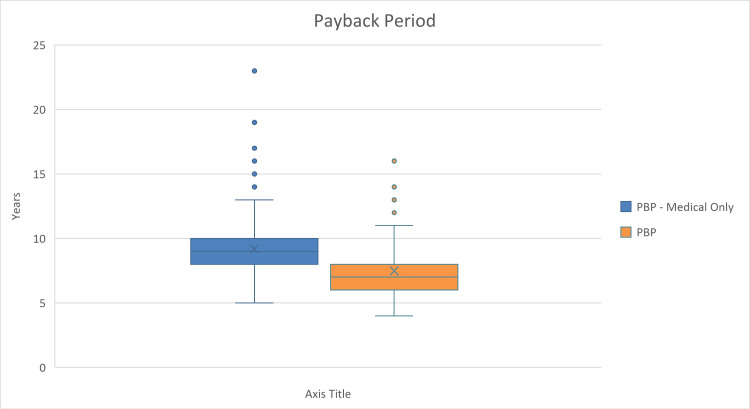
Probabilistic sensitivity analysis (PSA) box and whiskers plot showing payback period of investment in the device. PBP: payback period.

For the PSA, we evaluated how changes in the multiple inputs can affect the NPV. We included several parameters’ probability distributions to run 1,000 model iterations. The results of the PSA are presented in box and whisker plots to display the variability and central tendency of NPV, BCR, and payback period in the two distinct scenarios (Figures 2-4). The figures show that among the 1,000 iterations, the median and interquartile values for BCR and NPV show favorable scenarios of investment, while in less frequent scenarios, the investment decision may be unfavorable.

The blue boxes represent calculations of NPV, BCR, or payback period based solely on medical factors, where the median is shown by the mark (X), with the interquartile range indicating the middle 50% of the data. The whiskers extend to show the full range, excluding outliers, which are denoted by the individual points. The orange boxes illustrate the NPV, BCR, or payback period when both medical and non-medical factors are considered.

Maximum capacity scenario results

If the device is utilized to its maximum capacity, the facility can treat a total of 491 patients annually, including both international and local patients. This calculation is based on a facility with two rooms, each serving 25 patients per day, with the device operational for 275 days per year, and an average of 35,000 USD in revenue per patient.

In maximum capacity, from the societal perspective, the BCR is high at 1.80 and the NPV further reinforces this positive outcome at 53,274,913 JOD. The IRR stands at 32%. From the RMS perspective, the BCR is 1.58, the NPV is 38,475,811 JOD, and the IRR is 24%. The payback periods from both perspectives align at five years. The maximum capacity scenario details are shown in Table 5.

**Table 5:** Maximum capacity scenario results. JOD: Jordanian dinar.

Outcomes	Jordanian economy perspective (medical and non-medical revenues)		Royal Medical Services perspective (only medical revenues)	
	Value	Interpretation	Value	Interpretation
Benefit-cost ratio (BCR)	1.80	For each JOD spent, the project returns 1.8 JOD	1.58	For each JOD spent, the project returns 1.6 JOD
Net present value (NPV)	53,274,913	The project returns 53,274,913 JOD	38,475,811	The project returns 38,475,811 JOD
Internal rate of return (IRR)	32%	Annual rate of growth	24%	Annual rate of growth
Payback period	5	The payback period is in year 5	5	The payback period is in year 5

Number of patients required to reach break-even

The BCR represents the ratio of total benefits to total costs over a defined period. A BCR of 1.0 indicates that the device’s benefits are equal to its costs, meaning it has reached its break-even point. To reach break-even from the Jordanian holistic economy perspective, 249 patients are required annually, while from the RMS perspective, break-even could be reached at 291 patients.

## Discussion

PT is gaining wider recognition for its greater benefits over conventional photon therapy, particularly its ability to significantly reduce radiation doses to healthy tissues. Furthermore, it is the intervention of choice for several pediatric tumors, highlighting its role in contemporary cancer treatment approaches [24].

Investing in a PT facility in Jordan is a strategic move that not only reflects the country's key healthcare goals but also solidifies its status as a promising hub for medical tourism. However, investing in such an expensive intervention requires careful modeling and forecasting for potential costs and revenues. This is specifically important as the country takes significant steps toward integrating health technology assessment and evidence-based decision-making in its healthcare policies [25,26].

Several key factors position Jordan as a potential hub for medical tourism and advanced healthcare services [9]. The country has a well-established infrastructure for catering to international patients, including logistical support, language assistance, and hospitality services. Moreover, Jordan’s cultural and linguistic alignment with Arabic-speaking countries gives it a unique edge, as Arabic-speaking patients may find it easier to communicate and feel more comfortable in a familiar cultural setting. These factors collectively form a competitive advantage that strengthens Jordan’s position as a favorable destination for PT, potentially attracting a higher influx of medical tourists and increasing the overall financial viability of the investment in PT infrastructure [9].

Incorporating non-medical revenues into the financial analysis enhances the projected benefits of PT, suggesting that a comprehensive approach, considering both medical and non-medical revenue streams can maximize the project's profitability and reinforce Jordan’s reputation in medical tourism.

The model’s base case results provide insights into the financial viability of the investment. From the societal perspective, every JOD invested is expected to return 1.24 JOD in return. This is complemented by an NPV of around 15 million JOD, suggesting a substantial positive return. The IRR indicates a healthy annual rate of growth at 12%. Furthermore, the investment is expected to break-even in the seventh year, as indicated by the payback period.

From the healthcare payer perspective, only medical revenues are considered rendering the model less favorable. For each JOD spent, the investment is projected to return 1.1 JOD. The NPV in this scenario is 5 million JOD, which is significantly lower than that of the societal perspective, but still denotes a positive return. The IRR is estimated at 6%, reflecting a modest annual growth rate. Additionally, the payback period is longer, with the investment expected to break-even in year nine. This comparison underscores the enhanced financial benefits of considering both medical and non-medical revenues in the financial benefit-cost analysis of PT in Jordan.

The DSA results show that the population size, market uptake, revenue per session from non-Jordanian patients, and the number of sessions per patient significantly influence the NPV, with larger values in these categories correlating with an increase in NPV. This analysis underscores the importance of patient population, pricing strategies, and operational efficiencies in determining the financial viability of the investment.

The PSA results show that the NPV value considering only medical factors box presents a narrower range of values compared to the NPV value considering both medical and non-medical factors plot. The median values suggest that considering the broader economic effects reflects a higher median NPV. All median and interquartile range values of the NPV show positive values; however, there are outlier cases that could result in a negative NPV, presenting a risk of the investment being non-profitable, even with including non-medical factors. However, according to the PSA results, these outliers have a lower probability of occurring.

In Figure 3, the BCR box plots measure the relative profitability of the project. The plots show that the median BCR value is higher when considering the full spectrum of revenues. There is also a risk of BCR represented in some outlier values, which present a risk of the investment being not cost-beneficial; however, these are considered outlier values, while the median and interquartile ranges show a favorable BCR.

The payback period in Figure 4 shows the number of years to recover the initial investment. The plot illustrates that when considering only medical revenues, the median time to payback is longer, while a broader economic perspective could potentially reduce the time needed to recover the investment. The PSA suggests that the investment would be more financially viable when a comprehensive approach is adopted, compared to an approach that considers only the medical factors. Outlier values may show a risk of very long payback periods reaching up to more than 20 years with the medical revenues only included, and more than 15 years with all revenues included.

In the maximum capacity scenario, the Jordanian economy perspective shows an impressively high BCR value at 1.80. This indicates that for every JOD invested, a return of 1.8 JOD is expected. The NPV further reinforces this positive outcome, standing at more than 53 million JOD, indicating the project’s total net gain. The IRR at 32% signifies a robust annual rate of growth for the investment. From the RMS perspective assuming maximum utilization, the results are also favorable, albeit slightly lower than the societal perspective. For every JOD spent, the project is expected to return 1.6 JOD. The NPV is around 38 million JOD, indicating a significant return, though less than the societal perspective. The IRR is calculated at 24%, showing a strong annual growth rate. Additionally, the investment’s payback period from both perspectives is projected at five years, indicating a swift return on investment. Both perspectives demonstrate the financial viability of investing in PT in Jordan, with the broader economic perspective offering a slightly more advantageous return.

The break-even analysis shows the minimum number of patients required to ensure profitability. Without meeting these thresholds, the facility risks falling short of financial feasibility, underscoring the critical role of targeted marketing strategies, strong international partnerships, and policy initiatives to enhance medical tourism.

We validated the medical and ancillary costs provided by local experts with the data provided by a study reporting the hospital unit costs in Jordan [23] and found that most costs provided by the experts fell within the range provided by the study. To further test the model's sensitivity to varying cost inputs, we ran the model using the average cost values from this study. The model’s BCR was still more than 1 for both societal and public payer perspectives. This shows the robustness of the results, proving that the investment decision would not change, even if the unit costs were slightly varied during the upcoming period.

In Jordan, no research has been conducted to explore the benefit of PT as an investment or as a medical tourism attraction. This makes it challenging to compare our outcomes to other similar studies.

However, while searching for cost-benefit analyses of PT in other countries, we identified a recent systematic review that examined the methodology and quality of economic evaluations for PT in adult cancer. This study identified only seven studies that met the inclusion criteria, highlighting the scarcity of research in this field. None of those studies evaluated the effect of PT on medical tourism [27].

Another systematic review focused on the health economic evaluations of PT for lung cancer identified only four eligible studies, with no consideration of medical tourism [28].

The findings of this model are not generalizable to other countries or disease areas due to the significant differences in model inputs between countries. However, the methodology, model structure, and calculations can be adapted to another setting to create valid local results if required.

Decision-makers are expected to use the model results to assess and finalize the investment decision. While there is a risk of the investment being non-profitable, considering the broader benefits of medical tourism reduces this risk and justifies the investment. Enhancing decision-makers’ confidence can also be achieved through reducing uncertainty. Uncertainty surrounding the model findings can be reduced through innovative strategies. For instance, uncertainty in device utilization can be addressed by partnering with international organizations to direct their eligible patients to be treated in Jordan. Additional strategies include optimizing the workforce and negotiating better contract terms with the manufacturer for acquisition or maintenance costs or both.

Limitations

As we interpret our study results, it is important to keep in mind the limitations that must be considered. The local model inputs and estimations of patient populations were based on expert opinions. While this approach yielded valuable insights, it may not be as accurate as real-world data collection. To address this potential limitation and assure the credibility of the model, we conducted sensitivity analyses, which allowed us to consider a variety of outcomes and gain a deeper understanding of how our estimations may influence the overall results.

Several data inputs in the model are intended for and might apply only to the current year (e.g., salaries, device price, and facility utilities). Thus, if the model is used in another year, the current results will not be valid. Therefore, an option was added to adjust model inputs to accurately reflect the changes in years, time horizons, or costs.

Another limitation is the reliance on existing research to estimate the patient group that could benefit from PT, which restricts our assessment to the types and scopes of cancer treatments documented in current literature. As the body of evidence around PT continues to evolve with new studies being published about its application across various types of cancer, there is a potential to reconsider and possibly expand the group of patients identified as beneficiaries of this treatment modality. However, our estimations here are conservative.

The potential of the device was estimated assuming that no PT devices will be available in the Middle East region in the upcoming period. If another country has this device, it is likely that the target patient population will be divided by those two facilities, rendering the model results inaccurate. Therefore, before the investment decision, the decision-makers should make sure that no neighboring countries are willing to invest in a PT facility in the next period.

## Conclusions

The cost-benefit analysis concluded that investing in the PT facility in Jordan is cost-beneficial, especially from a societal perspective. The facility is expected to generate medical revenues and attract international patients, reinforcing Jordan's vision of being a leader in medical tourism. As per the model results, if medical tourism benefits are not considered, investing in the facility remains a cost-beneficial decision; however, benefits are reduced compared to scenarios that include medical tourism revenues. The model results carry some degree of uncertainty, primarily related to the number of patients who are expected to utilize the device. Additionally, other uncertainties arise from the number of sessions per patient, operational costs, and marketing costs. Sensitivity analyses show that results are robust to varying several inputs, and across different scenarios. However, in extreme scenarios, such as significantly lower device utilization, significantly reduced average number of sessions, or substantially increased operational costs, the investment decision is at risk of being less favorable. It is important to implement strategies to reduce these uncertainties before making an investment decision.

Despite the challenges, Jordan's established reputation in medical tourism, strategic geographical location, and advanced healthcare infrastructure provide a strong foundation to maximize the potential of PT. By leveraging these advantages, Jordan can position itself as a regional leader in advanced cancer treatment, enhancing both its healthcare system and economic growth. Future research is recommended to provide more comprehensive and high-quality inputs for conducting better-quality models and creating robust analyses. Additionally, we encourage developing similar models to support decision-makers in making evidence-based investment decisions.

## Appendices

**Table 6:** Experts' questionnaire.

Will the construction be in a greenfield or within an existing radiotherapy (RT) facility?
…………………………………………………………………………………………
What is the number of rooms in the facility (rooms for treatment)?
…………………………………………………………………………………………
What would the cost of the proton therapy equipment and its installation be? (From the manufacturer)
…………………………………………………………………………………………
What is the cost of establishing the other clinical support rooms? if there would be any of them (e.g., exam rooms, waiting area, set-up rooms, check-in, consult rooms, on-treatment visit (OTV) rooms, simulation, mold preparation, and guidance to a treatment room being used for a patient’s specific condition).
……………………………………………………………………………………………
What is the expected cost of the other equipment (other than the main equipment)? E.g.:
Other imaging equipment (MRI, PET, and any other considered necessary)
Laboratory (if needed)
Administrative equipment (computers, printers,…etc.)
Anesthesia equipment
Camera system for 3D surface scanning
……………………………………………………………………………………………
What is the expected annual cost of consumables?
……………………………………………………………………………………………
What is the expected annual cost of maintenance (from the manufacturer)?
……………………………………………………………………………………………
What is the expected annual cost of training?
……………………………………………………………………………………………
What is the expected price per session for non-Jordanians (price charged to the payers)?
……………………………………………………………………………………………
What are the expected working hours per day?
……………………………………………………………………………………………
What is the expected number of working days per year?
……………………………………………………………………………………………
What is the estimated number of Jordanians who travel abroad to receive proton therapy per year and their cost?
……………………………………………………………………………………………
Please provide us with the indication mix to be treated by the facility (accepted indications to be treated).
……………………………………………………………………………………………
What will be the average number of sessions per patient (average according to the treatment protocol of the facility)?
……………………………………………………………………………………………
What is the estimated number of Jordanians who may receive treatment in the facility in the future (for example, 2024)?
Number
Price per session (price charged to the payers).
……………………………………………………………………………………………
For the expected medical staff required per room (please indicate the number and monthly salary). Please make any modifications required.
Physician (radiation oncologist)
Nurse
Nurse aids
Radiation therapists
Dosimetrist
Medical physicist
Anesthesia specialist
……………………………………………………………………………………………
What is the cost of establishing office space for staff (e.g.: rooms, etc.)?
……………………………………………………………………………………………
What is the expected annual cost of insurance?
……………………………………………………………………………………………
What is the expected annual cost of marketing?
……………………………………………………………………………………………
What is the expected annual cost of electricity?
……………………………………………………………………………………………
Please provide us with the available data about the non-medical expenditure of medical tourists and their companions in Jordan.
Medical tourists
Companions
……………………………………………………………………………………………
For the expected administrative staff required (please indicate the number and monthly salary). Please make any modifications required.
Secretary
Information and technology support
Financial department
Human resources
……………………………………………………………………………………………

**Table 7:** Experts' responses to the questionnaire. RT: radiotherapy; JOD: Jordanian dinar; OTV: on-treatment visit; MRI: magnetic resonance imaging; PET: positron emission tomography; CT: computed tomography; CBC: complete blood count; USD: United States dollar; OIS: oncology information system; TPS: treatment planning system.

Questions	Expert answers
Will the construction be in a greenfield or within an existing radiotherapy (RT) facility?	Greenfield
What is the number of rooms in the facility (rooms for treatment)?	2 rooms
What would the cost of the proton therapy equipment and its installation be? (From the manufacturer)	Cost per room ranges between 20 and 25 million JOD, including construction, proton therapy system, treatment plan, transport, and data collection proton therapy systems (OIS/TPS), greenfield cost, proton therapy system building unit, distributed costs
What is the cost of establishing the other clinical support rooms? If there would be any of them (e.g., exam rooms, waiting area, set-up rooms, check-in, consult rooms, on-treatment visit (OTV) rooms, simulation, mold preparation, and guidance to a treatment room being used for a patient’s specific condition)	“Mold preparation” is not needed
What is the expected cost of the other equipment (other than the main equipment)? (E.g., other imaging equipment (MRI, PET, and any other considered necessary), laboratory (if needed), administrative equipment (computers, printers,…etc.), anesthesia equipment, and camera system for 3D surface scanning)	CT simulation and camera system for 3D surface scanning are included in the cost of the proton therapy installment. There is no need for PET, MRI, or other scans as it is available in the medical centers. For lab tests, only CBC is needed for anesthesia, and only simple equipment is needed. So, the cost in this question is negligible.
What is the expected annual cost of consumables?	No information is available for all consumables related to the proton therapy, but fixation device costs can be taken into consideration, and costs range from 70 to 100 JOD per patient. Given that not all patients need it, only children.
What is the expected annual cost of maintenance (from the manufacturer)?	Usually, maintenance cost is covered by the manufacturer between 7 and 10 years based on expert opinions. The maintenance cost ranged from 800,000 JOD per year.
What is the expected annual cost of training?	The training cost is usually overlooked in the calculations and is typically included in the bid.
What is the expected price per session for non-Jordanians (price charged to the payers)?	According to international trials, the estimated cost of a complete treatment journey ranges from 30,000 USD, which means one session cost ranges from 1,000 to 1,500 USD.
What are the expected working hours per day?	12-14 hours. Device capacity: approximately 50 patients per day for the two rooms.
What is the expected number of working days per year?	275 days
What is the estimated number of Jordanians who travel abroad to receive proton therapy per year and their cost?	Information not available
Please provide us with the indication mix to be treated by the facility (accepted indications to be treated).	Pediatric: all solid tumors. Adult: brain tumor, retroperitoneal tumor, skull base tumor, and sarcoma.
What will be the average number of sessions per patient (average according to the treatment protocol of the facility)?	The patient needs approximately 25-30 sessions.
What is the estimated number of Jordanians who may receive treatment in the facility in the future (for example 2024)? Number and price per session (price charged to the payers)	Children: range from 135 to 150 cases per year. Adults: range from 300 to 400 cases per year.
For the expected medical staff required per room (please indicate the number and monthly salary). Please make any modifications required. (Physician (radiation oncologist), nurse, nurse aids, radiation therapists, dosimetrist, medical physicist, and anesthesia specialist)	Physician (radiation oncologist): 4; salary per one per month: 4,000 JOD. Nurse: 4; salary per one per month: 1,500 JOD. Nurse aids: 4; salary per one per month: 600 JOD. Radiation therapists: 12; salary per one per month: 800 JOD. Dosimetrist: 2; salary per one per month: 1,000 JOD. Medical physicist: 4; salary per one per month: 800 JOD. Anesthesia specialist: 2; salary per one per month: 800 JOD.
What is the cost of establishing office space for staff (e.g.: rooms, etc.)?	Included above
What is the expected annual cost of insurance?	The company is responsible for shipping and installment.
What is the expected annual cost of marketing?	Information not available
What is the expected annual cost of electricity?	Should be calculated based on average cost per patient per treatment course and cost per kWh.
Please provide us with the available data about the non-medical expenditure of medical tourists and their companions in Jordan.	Estimated expenses of one household (patient with dependent) range from 3,000 to 5,000.
For the expected administrative staff required (please indicate the number and monthly salary). Please make any modifications required. (Secretary, information and technology support, financial department, and human resources)	Secretary: 2; salary per one per month: 450 JOD. Information and technology support: 1; salary per one per month: 600 JOD. Financial department: 2; salary per one per month: 800 JOD. Human resources: 10; salary per one per month: 300 JOD.

**Table 8:** Parameters involved in DSA and PSA. LoS: length of stay; IT: information and technology; kWh: kilowatt per hour.

Parameters	Distribution
Number of rooms	Gamma
Maintenance costs start at year	Normal
Capacity of patients per day per room	Normal
Device working days per year	Gamma
Number of sessions per patient	Gamma
Length of treatment in days	Gamma
Average LoS of international in days	Gamma
Physicians (radiation oncologist) required per room	Gamma
Nurses required per room	Gamma
Nurse aids required per room	Gamma
Radiation therapist required per room	Gamma
Dosimetrist required per room	Gamma
Medical physicist required per room	Gamma
Anesthesia specialist required per room	Gamma
Secretary required	Gamma
Information and technology (IT) specialist support required	Gamma
Financial department employee required	Gamma
Human resources (workers) required	Gamma
Model costs	
Device related costs	
Equipment and installation per room	Gamma
Other equipment	Gamma
Training (annual)	Gamma
Consumables per patient	Gamma
Maintenance cost (annual)	Gamma
Marketing (annual)	Gamma
Cost of electricity per kWh	Gamma
Device electricity usage per treatment course per patient (kWh)	Gamma
Medical staff costs (salary per month)	
Physician (radiation oncologist)	Gamma
Nurse	Gamma
Nurse aid	Gamma
Radiation therapist	Gamma
Dosimetrist	Gamma
Medical physicist	Gamma
Anesthesia specialist	Gamma
Non-medical staff costs (salary per month)	
Secretary	Gamma
Information and technology (IT) support specialist	Gamma
Financial department employee	Gamma
Human resources (workers)	Gamma
Treatment abroad	
Cost of treatment outside Jordan	Gamma
Model benefits	
Revenue per session non-Jordanian	Gamma
Revenue per session Jordanian	Gamma
Daily living cost per person with one dependent	Gamma
Population	
Jordanian	
Eligible population	Normal
Market uptake	Normal
Patients who receive treatment outside of Jordan	Normal
Proportion of patients treated at RMS	Beta
Non-Jordanian	
Eligible population	Normal
Market uptake	Normal

## References

[1] (2024). American Cancer Society. Proton beam therapy for prostate cancer still needs studying. https://www.cancer.org/research/acs-research-highlights/prostate-cancer-research-highlights/treatment-studies/proton-beam-therapy-for-prostate-cancer-still-needs-studying.html#:~:text=We%20need%20clinical%20trials%20for,to%20quality%20and%20affordable%20care..

[2] Eaton BR, MacDonald SM, Yock TI, Tarbell NJ (2015). Secondary malignancy risk following proton radiation therapy. Front Oncol.

[3] Liu H, Chang JY (2011). Proton therapy in clinical practice. Chin J Cancer.

[4] Chen YH, Blommestein HM, Klazenga R, Uyl-de Groot C, van Vulpen M (2023). Costs of newly funded proton therapy using time-driven activity-based costing in the Netherlands. Cancers (Basel).

[5] Álvarez SIP, Ruiz FJL, Magos FM, García AM (2023). Proton therapy in lower-middle-income countries: from facts and reality to desire, challenges and limitations. Proton Therapy - Current Status and Future Directions.

[6] (2023). Particle Therapy Co-Operative Group. Particle therapy facilities in clinical operation. https://www.ptcog.site/index.php/facilities-in-operation-public.

[7] Safavi AH, Freeman C, Cheng S (2023). Proton therapy in Canada: toward universal access and health equity with a publicly funded facility. Int J Radiat Oncol Biol Phys.

[8] Elsayed Z, Lalya I, AlHussain H, Mula-Hussain L (2022). Radiation therapy in Arab world. Cancer in the Arab World.

[9] Anshasi RJ, Alsyouf A, Alhazmi FN (2022). Jordan as a medical hotspot: views on medical tourism. Int J Prof Bus Rev.

[10] (2024). Al-Kindi Hospital. Medical tourism in Jordan. https://al-kindihospital.com/en/medical-tourism-in-jordan/.

[11] Newhauser WD, Zhang R, Jones TG (2015). Reducing the cost of proton radiation therapy: the feasibility of a streamlined treatment technique for prostate cancer. Cancers (Basel).

[12] Mailhot Vega RB, Mohammadi H, Patel SI (2022). Establishing cost-effective allocation of proton therapy for patients with mediastinal Hodgkin lymphoma. Int J Radiat Oncol Biol Phys.

[13] Li G, Qiu B, Huang YX (2020). Cost-effectiveness analysis of proton beam therapy for treatment decision making in paranasal sinus and nasal cavity cancers in China. BMC Cancer.

[14] Akash M, Salem A (2023). Proton beam therapy in the Middle East: modelling demand and health economic cost. Radiat Oncol.

[15] Al-Sayaideh A, Nimri O, Arqoub K, Al-Zaghal M, Halasa W (2016). Cancer Incidence in Jordan - 2012. Jordan Cancer Registry.

[16] Rihani R, Jeha S, Nababteh M, Rodriguez-Galindo C, Mansour A, Sultan I (2023). The burden and scope of childhood cancer in displaced patients in Jordan: the King Hussein Cancer Center and Foundation Experience. Front Oncol.

[17] Yan S, Ngoma TA, Ngwa W, Bortfeld TR (2023). Global democratisation of proton radiotherapy. Lancet Oncol.

[18] Mousa AG, Bishr MK, Mula-Hussain L, Zaghloul MS (2019). Is economic status the main determinant of radiation therapy availability? The Arab world as an example of developing countries. Radiother Oncol.

[19] Kim J, Wells C, Khangura S (2017). Proton Beam Therapy for the Treatment of Cancer in Children and Adults: A Health Technology Assessment. Ottawa (ON): Canadian Agency for Drugs and Technologies in Health.

[20] (2024). Texas Center for Proton Therapy. Common questions about proton therapy. https://www.texascenterforprotontherapy.com/proton-therapy/common-questions-about-proton-therapy.

[21] Dvorak T, Meeks S, Dvorak L (2023). Evaluating carbon footprint of proton therapy based on power consumption and possible mitigation strategies. Int J Radiat Oncol Biol Phys.

[22] (2023). Jordan electricity prices. https://www.globalpetrolprices.com/Jordan/electricity_prices/.

[23] Hammad EA, Alabbadi I, Taissir F, Hajjwi M, Obeidat NM, Alefan Q, Mousa R (2022). Hospital unit costs in Jordan: insights from a country facing competing health demands and striving for universal health coverage. Health Econ Rev.

[24] Yuan TZ, Zhan ZJ, Qian CN (2019). New frontiers in proton therapy: applications in cancers. Cancer Commun (Lond).

[25] Almomani E, Alabbadi I, Fasseeh A (2021). Implementation road map of health technology assessment in middle-income countries: the case of Jordan. Value Health Reg Issues.

[26] Almomani E, Hammad EA, AlQutob R (2022). Capacity building for health technology assessment in Jordan: institutionalization and its use in pricing and reimbursement decisions. Value Health Reg Issues.

[27] Jones DA, Smith J, Mei XW (2020). A systematic review of health economic evaluations of proton beam therapy for adult cancer: appraising methodology and quality. Clin Transl Radiat Oncol.

[28] Li CC, Lin YC, Liang JA, Chao KS, Hsia TC, Chien CR (2023). Health economic evaluation of proton therapy for lung cancer: a systematic review. Int J Environ Res Public Health.

